# Dampened activity of ryanodine receptor channels in mutant skeletal muscle lacking TRIC‐A

**DOI:** 10.1113/JP273550

**Published:** 2017-05-23

**Authors:** Sam El‐Ajouz, Elisa Venturi, Katja Witschas, Matthew Beech, Abigail D. Wilson, Chris Lindsay, David Eberhardt, Fiona O'Brien, Tsunaki Iida, Miyuki Nishi, Hiroshi Takeshima, Rebecca Sitsapesan

**Affiliations:** ^1^ Department of Pharmacology University of Oxford Oxford UK; ^2^ Department of Chemistry University of Oxford Oxford UK; ^3^ Graduate School of Pharmaceutical Sciences Kyoto University Kyoto Japan

**Keywords:** Ca^2+^ release, ryanodine receptor, sarcoplasmic reticulum

## Abstract

**Key points:**

- The role of trimeric intracellular cation (TRIC) channels is not known, although evidence suggests they may regulate ryanodine receptors (RyR) via multiple mechanisms. We therefore investigated whether *Tric‐a* gene knockout (KO) alters the single‐channel function of skeletal RyR (RyR1).
- We find that RyR1 from *Tric‐a* KO mice are more sensitive to inhibition by divalent cations, although they respond normally to cytosolic Ca^2+^, ATP, caffeine and luminal Ca^2+^.
- In the presence of Mg^2+^, ATP cannot effectively activate RyR1 from *Tric‐a* KO mice.
- Additionally, RyR1 from *Tric‐a* KO mice are not activated by protein kinase A phosphorylation, demonstrating a defect in the ability of β‐adrenergic stimulation to regulate sarcoplasmic reticulum (SR) Ca^2+^‐release.
- The defective RyR1 gating that we describe probably contributes significantly to the impaired SR Ca^2+^‐release observed in skeletal muscle from *Tric‐a* KO mice, further highlighting the importance of TRIC‐A for normal physiological regulation of SR Ca^2+^‐release in skeletal muscle.

**Abstract:**

The type A trimeric intracellular cation channel (TRIC‐A) is a major component of the nuclear and sarcoplasmic reticulum (SR) membranes of cardiac and skeletal muscle, and is localized closely with ryanodine receptor (RyR) channels in the SR terminal cisternae. The skeletal muscle of *Tric‐a* knockout (KO) mice is characterized by Ca^2+^ overloaded and swollen SR and by changes in the properties of SR Ca^2+^ release. We therefore investigated whether RyR1 gating behaviour is modified in the SR from *Tric‐a* KO mice by incorporating native RyR1 into planar phospholipid bilayers under voltage‐clamp conditions. We find that RyR1 channels from *Tric‐a* KO mice respond normally to cytosolic Ca^2+^, ATP, adenine, caffeine and to luminal Ca^2+^. However, the channels are more sensitive to the inactivating effects of divalent cations, thus, in the presence of Mg^2+^, ATP is inadequate as an activator. Additionally, channels are not characteristically activated by protein kinase A even though the phosphorylation levels of Ser2844 are similar to controls. The results of the present study suggest that TRIC‐A functions as an excitatory modulator of RyR1 channels within the SR terminal cisternae. Importantly, this regulatory action of TRIC‐A appears to be independent of (although additive to) any indirect consequences to RyR1 activity that arise as a result of K^+^ fluxes across the SR via TRIC‐A.

## Key points

- The role of trimeric intracellular cation (TRIC) channels is not known, although evidence suggests they may regulate ryanodine receptors (RyR) via multiple mechanisms. We therefore investigated whether *Tric‐a* gene knockout (KO) alters the single‐channel function of skeletal RyR (RyR1).
- We find that RyR1 from *Tric‐a* KO mice are more sensitive to inhibition by divalent cations, although they respond normally to cytosolic Ca^2+^, ATP, caffeine and luminal Ca^2+^.
- In the presence of Mg^2+^, ATP cannot effectively activate RyR1 from *Tric‐a* KO mice.
- Additionally, RyR1 from *Tric‐a* KO mice are not activated by protein kinase A phosphorylation, demonstrating a defect in the ability of β‐adrenergic stimulation to regulate sarcoplasmic reticulum (SR) Ca^2+^‐release.
- The defective RyR1 gating that we describe probably contributes significantly to the impaired SR Ca^2+^‐release observed in skeletal muscle from *Tric‐a* KO mice, further highlighting the importance of TRIC‐A for normal physiological regulation of SR Ca^2+^‐release in skeletal muscle.

**Abbreviations**
DM: *n*‐decyl‐β‐d‐maltopyranoside
ER/SR: endoplasmic/sarcoplasmic reticulum
KO: knockout
MS: mass spectrometry
pdf: probability density function
PKA: protein kinase A
PP1: protein phosphatase 1
RyR: ryanodine receptor
SR: sarcoplasmic reticulum
TRIC: trimeric intracellular cation channel
WT: wild‐type

## Introduction

There are two subtypes of trimeric intracellular cation channel (TRIC), termed TRIC‐A and TRIC‐B, and both are found on the endoplasmic/sarcoplasmic reticulum (ER/SR) and the nuclear membranes of most cell types (Yazawa *et al*. 2007). The conductance and gating properties of purified recombinant TRIC channels reconstituted into artificial membranes are similar to those of the monovalent cation selective SR K^+^ channels first observed from preparations of isolated rabbit skeletal SR vesicles (Labarca *et al*. 1980; Yazawa *et al*. 2007; Pitt *et al*. 2010). The TRIC channels have been shown to be trimeric in structure, each formed from three individual monomers of ∼30 kDa in molecular mass (Yazawa *et al*. 2007; Kasuya *et al*. 2016; Yang *et al*. 2016). It was assumed that the SR K^+^ channel fulfils the essential role of a counter‐ion pathway, allowing rapid charge compensation for the SR Ca^2+^ release via ryanodine receptor (RyR) channels (Miller & Rosenberg 1979; Somlyo *et al*. 1981; Fink & Veigel 1996). It was subsequently suggested that the RyR channels may be able to pass most or all of their own counter‐current (Gillespie & Fill 2008; Gillespie *et al*. 2009). If this is so, then the necessity for the SR K^+^ channel to pass counter‐ion flux is not as critical as first assumed, although equilibration of K^+^ across the SR will still be important. Gene knockout (KO) studies, however, demonstrate that TRIC is essential for the normal functioning of many tissues. For example, the *Tric‐a*/*Tric‐b* double KO mouse dies in heart failure before birth (Yazawa *et al*. 2007). The *Tric‐b* KO mouse dies immediately after birth in respiratory failure and the *Tric‐a* KO mouse exhibits an abnormal SR ultrastructure and unstable contractile behaviour under stress in skeletal muscle (Yamazaki *et al*. 2009; Zhao *et al*. 2010). More recently, mutations in *TRIC‐B* have been associated with the disease osteogenesis imperfecta (Volodarsky *et al*. 2013; Rubinato *et al*. 2014). The absolute requirement in some tissues for the presence of TRIC perhaps indicates additional roles of the TRIC channels in addition to their capacity to act as pathway for monovalent cation flux across the SR. Investigation of TRIC:RyR stoichiometry in various tissues indicates that, in excitable tissues such as cardiac and skeletal muscle, the SR is packed with many more TRIC‐A channels than RyR and TRIC‐B channels (Pitt *et al*. 2010; Zhao *et al*. 2010). RyR and TRIC channels have not been co‐purified in previous biochemical studies (Yazawa *et al*. 2007); however, reversible protein–protein interactions between the densely packed ion channels in the SR may provide an important regulatory influence on RyR activity and SR Ca^2+^ release. Indeed, a protein termed SPR‐27 but subsequently discovered to be the same protein as TRIC‐A was previously suggested to form part of the RyR macromolecular complex (Bleunven *et al*. 2008). Because the *Tric‐a* KO mouse survives until adulthood, we isolated SR membranes from the mature skeletal muscle and incorporated them into bilayers to investigate whether the gating or conductance of the RyR channels are modified by the absence of TRIC‐A. The results obtained show that the RyR from *Tric‐a* KO mice exhibit modified gating properties that prevent the channels from responding normally to activators such as ATP or to phosphorylation by protein kinase A (PKA).

## Methods

### Ethical approval

All experiments in the present study were conducted with the approval of the Animal Research Committee in accordance with the regulations on animal experimentation at Kyoto University (Agreement no. 11‐6).

### Isolation of membrane fractions from mouse skeletal muscle

Isolated membrane vesicles were prepared from wild‐type (WT) and *Tric‐a* KO mouse skeletal muscle using methods described previously (Venturi *et al*. 2013) with some modifications. Mouse skeletal muscle was dissected and snap‐frozen in liquid N_2_. Frozen tissue was pulverized and finely homogenised in a buffer containing 300 mm sucrose and 20 mm PIPES (pH 7.4) and supplemented with a protease inhibitor cocktail (Sigma‐Aldrich, Poole, UK), 1 mm phenylmethane sulphonyl fluoride and 2.5 mm dithiothreitol. The tissue homogenate was centrifuged at 6000 *g* for 20 min at 4°C. The supernatant obtained was collected and the pellets were re‐homogenized, resuspended in the same buffer and centrifuged at 6000 *g* for 20 min at 4°C. The supernatants obtained were filtered through a cheesecloth and spun at 100 000 *g* for 1 h at 4°C. The pellets, containing the membrane fractions, were resuspended in 400 mm sucrose, 5 mm HEPES, 2.5 mm DTT (pH 7.2), aliquoted, snap‐frozen in liquid N_2_ and stored at −80°C. Isolated membrane fractions were used in single‐channel and [^3^H]ryanodine binding experiments.

### Purification of TRIC‐A

A stable Chinese hamster ovary cell line overexpressing mouse TRIC‐A was generated. The cDNA encoding the full‐length mouse TRIC‐A was fused with a PA‐tag at the N‐terminal and subcloned into the pcDNA3 expression vector (Invitrogen, Carlsbad, CA, USA). Cells expressing TRIC‐A were cultured in α‐MEM (Gibco, Gaithersburg, MD, USA) with 10% FBS (Sigma), 1:200 penicillin–streptomycin (Sigma) and 200 μg ml^−1^ G418 (Sigma). Cells were cultured in 25 × 175 cm^2^ flasks, collected and homogenized with a dounce homogenizer in hypotonic buffer containing 10 mm HEPES (pH 7.4). Solubilisation was achieved by the addition of an equal volume of 2x binding buffer containing 0.5 m sucrose, 0.6 m NaCl, 10 mm HEPES (pH 7.4) and 2% (w/v) *n*‐decyl‐β‐d‐maltopyranoside (DM) to the cell lysate followed by a re‐homogenization step. Insoluble material was pelleted by a high‐speed centrifugation step (200 000 *g* for 30 min at 4°C) when the supernatant containing soluble proteins was collected. The supernatant was diluted to reduce the detergent concentration to 0.5% by adding an equal volume of 1x binding buffer (0.25 m sucrose, 0.3 m NaCl, 10 mm HEPES, pH 7.4). Anti‐PA tag antibody beads (Wako Chemicals GmbH, Neuss, Germany) were added to the supernatant and the mixture was then incubated with continuous stirring for 2 h at 4°C. The beads were then transferred into a centrifuge column (Thermo Fisher Scientific, Waltham, MA, USA) and washed five times in a washing buffer containing 10% (v/v) glycerol, 0.4 m NaCl, 1 mm EDTA, 20 mm Tris‐HCl, pH 7.4 and 0.1% DM. Fractions containing purified TRIC‐A proteins were eluted by supplementing the washing buffer with 0.2 mg ml^−1^ PA‐tag peptide (Wako Chemicals GmbH). All purification steps were carried out at 4°C. The buffers used for the purification were supplemented with 1 mm DTT and a protease inhibitor cocktail (Sigma). Western blot using an anti‐PA tag antibody (dilution 1:2000; Wako Chemicals GmbH) was used to confirm protein purification and enrichment.

### Reconstitution of purified TRIC‐A into liposomes

Phosphatidylcholine (Avanti Polar Lipids, Alabaster, AL, USA) in chloroform solution was dried under a nitrogen stream, resuspended in reconstitution buffer (100 mm NaCl, 20 mm HEPES, pH 7.4) at a concentration of 10 mg ml^−1^ and sonicated until the lipids formed a cloudy homogeneous suspension. Liposomes were disrupted by adding 35 mm 3‐[(3‐cholamidopropyl)dimethylammonio]‐1‐propanesulphonate. Purified TRIC‐A protein was added to the clear suspension at a protein to lipid ratio of 1:1 (v:v). An equal volume of washing buffer containing 0.1% DM was added to the lipid to produce empty control liposomes. The mixture was then dialysed in a 10 kDa‐cut‐off Slide‐A‐Lyzer cassette (Thermo Fisher Scientific) against 1 litre of reconstitution buffer for 6 h with buffer exchange every hour at 4°C. Liposomes containing TRIC‐A or empty liposomes were added to the cytosolic side of RyR1 channels from *Tric‐a* KO mice gating in bilayers with Ca^2+^ as the permeant ion (see below).

### Single‐channel recordings

Single‐channel recordings of RyR channels obtained from WT and *Tric‐a* KO skeletal muscles were performed as described previously (Sitsapesan *et al*. 1991). RyR current fluctuations were recorded under voltage clamp conditions using K^+^ or Ca^2+^ as the permeant ion. Isolated membrane vesicles containing RyR channels always incorporated in a fixed orientation such that the *cis* chamber corresponded to the cytosol, whereas the *trans* chamber corresponded to the SR lumen. For experiments with Ca^2+^ as the permeant ion, recording solutions were 250 mm HEPES, 80 mm Tris and 10 μm free Ca^2+^ (pH 7.2) on the *cis* side and 250 mm glutamic acid and 10 mm HEPES (pH to 7.2) with Ca(OH)_2_ (free [Ca^2+^] ∼50 mm) on the *trans* side of the bilayer. The *trans* chamber was voltage clamped at ground. For experiments with K^+^ as the permeant ion, symmetrical solutions of 210 mm KPIPES (pH 7.2) were used and luminal and cytosolic free [Ca^2+^] was adjusted as required. PKA‐dependent phosphorylation of RyR was achieved by incubating the cytosolic side of the channels with 10 units of the catalytic subunit of PKA (Sigma‐Aldrich) in the presence of 10 μm free Ca^2+^, 3 mm ATP and 1 mm free Mg^2+^ for 10 min. Single RyR channels were treated with 5 units of protein phosphatase 1 (PP1) (New England Biolabs, Beverly, MA, USA) in presence of Mn^2+^ for 10 min. After the PKA or PP1 incubation, the cytosolic chamber was washed back to control conditions. Experiments were performed at room temperature (22 ± 2°C). The free [Ca^2+^] and pH of the solutions were maintained constant during the experiment and were determined using a Ca^2+^ electrode (Orion 93‐20; Thermo Fisher Scientific) and a Ross‐type pH electrode (Orion 81‐55; Thermo Fisher Scientific) as described previously (Sitsapesan *et al*. 1991).

### Single‐channel analysis

Single‐channel recordings were digitized at 20 kHz and recorded on a computer hard drive using pClamp (Molecular Devices, Sunnyvale, CA, USA). Before idealization, traces were filtered at 800 Hz (−3 db) in experiments where Ca^2+^ was the permeant ion or at 4 kHz where K^+^ was the permeant ion. The open and closed channel levels were assessed using manually controlled cursors. Open probability (Po) was determined over 3 min of continuous recording using the 50% threshold method (Colquhoun & Sigworth 1983) at 0 mV, when Ca^2+^ was the permeant ion or at potentials relative to ground in K^+^‐containing solutions. Lifetime distributions were calculated from idealizations where only a single channel was gating in the bilayer. Events shorter than 1 ms (where Ca^2+^ was the permeant ion) or 0.2 ms (where K^+^ was the permeant ion) were stripped from the idealized event sequences using Clampfit, version 10.2 (Molecular Devices). Individual time constants were fitted with an exponential log probability density function (pdf) in Clampfit, using maximum‐likelihood fitting (Colquhoun & Sigworth 1983). The optimal number of time constants for each distribution was determined using a log‐likelihood ratio test at a confidence level of *P* = 0.95 (Blatz & Magleby 1986).

### RyR1 immunoprecipitation and immunoblotting

RyR1 was immunoprecipitated from 400 μg of mouse skeletal mixed membrane preparations using an anti‐RyR antibody (34C; Abcam, Cambridge, UK) and Protein G Dynabeads (Life Technologies, Oslo, Norway) by overnight incubation at 4°C with continuous mixing in 0.4 ml of homogenization buffer [20 mm HEPES, 150 mm NaCl, 5 mm EDTA, 20% (v/v) glycerol, protease inhibitors (Roche Diagnostics Limited, Burgess Hill, UK), Triton‐X 0.5%, pH 6.8]. Protein immunocomplexes were separated magnetically (DynaMag‐2 magnet; Thermo Fisher) and beads were washed three times with homogenization buffer. For PKA phosphorylation of RyR1, beads were incubated with 1 U of the catalytic subunit of PKA (Sigma‐Aldrich) per μg protein for 10 min at 37°C in a solution containing 50 mm HEPES, 16 mm Tris, 5 mm Mg^2+^ and 5 mm NaF (pH 7.2). After PKA treatment, the supernatant was removed by magnetic separation and samples were resuspended in 50 μl of Laemmeli sample buffer containing 5% β‐mercaptoethanol and incubated at 95°C for 5 min. Samples were then used for western blotting as described previously (Carter *et al*. 2011). Immunoblots were probed with anti‐RyR antibody (34C; dilution 1:1000) and Phospho‐(Ser/Thr) PKA Substrate Antibody #9621 (dilution 1:1000; Cell Signaling Technology, Leiden, The Netherlands). RyR1 protein and phosphorylation levels were quantified by densitometry.

### Mass spectrometry (MS) methods

Microsomes from WT and *Tric‐a* KO mouse skeletal muscle were treated with PKA as described previously (Carter *et al*. 2011). Microsomal proteins were separated on a 6% SDS‐PAGE and either stained with Coomassie Brilliant Blue for visualization or transferred to a nitrocellulose membrane and probed with RyR1 antibody as described above. The corresponding bands containing RyR1 were cut from the Coomassie Brilliant Blue stained gel and subjected to in‐gel tryptic digestion for MS analysis as described previously (Shevchenko *et al*. 2007). The peptides generated were then separated by nanoflow reversed‐phase liquid chromatography coupled to Q Exactive Hybrid Quadrupole‐Orbitrap mass spectrometer (Thermo Fisher Scientific). Peptides were loaded on a C18 PepMap100 pre‐column (inner diameter 300 μm × 5 mm, 3 μm C18 beads; Thermo Fisher Scientific) and separated on a 50 cm reversed‐phase C18 column (inner diameter 75 μm, 2 μm C18 beads). Separation was conducted with a linear gradient of 7–30% of B for 30 min at a flow rate of 200 nl min^−1^ (A: 0.1% formic acid, B: 0.1% formic acid in acetonitrile). All data were acquired in a data‐dependent mode, automatically switching from MS to collision‐induced dissociation MS/MS on the top 10 most abundant ions with a precursor scan range of 350–1650 *m*/*z*. MS spectra were acquired at a resolution of 70 000 and MS/MS scans at 17 000. Dynamic exclusion was enabled with an exclusion duration of 40 s. The raw data files generated were processed using MaxQuant, version 1.5.0.35, integrated with the Andromeda search engine as described previously (Cox & Mann 2008; Cox *et al*. 2011). The MS/MS spectra were searched against the mouse proteome (UniProt 2013/04/03), precursor mass tolerance was set to 20 ppm with variable modifications defined as phosphorylation (S, T and Y). Enzyme specificity was set to trypsin with a maximum of two missed cleavages. Protein and peptide spectral matches false discovery rate was set at 0.01 and a minimum score of 40 and localization probability of > 0.7 for phosphopeptides. Match between runs was applied. The ratio of phosphorylated (S2844) to unphosphorylated KISQTAQTYDPR peptide intensities was calculated for three biological replicates of WT and *Tric‐a* KO under control and PKA treatment.

### [^3^H]ryanodine binding

Binding of [^3^H]ryanodine (PerkinElmer Inc., Waltham, MA, USA) to skeletal membrane vesicles was measured at 37°C for 90 min with constant shaking in buffer consisting of 200 μg protein ml^–1^, 5 nm [^3^H]ryanodine, 250 mm KCl, 25 mm PIPES and 200 μm AEBSF (pH 7.2). RyR1 channel modulators Ca^2+^, Mg^2+^, caffeine and adenine were included in specific experiments as described where appropriate. Non‐specific binding was determined in the presence of a 1000‐fold excess unlabelled ryanodine. Bound and free ligand were separated by rapid filtration through Whatman GF/B glass microfibre filters (GE Healthcare Life Sciences, Little Chalfont, UK). [^3^H]ryanodine retained in filters was quantified by liquid scintillation spectrometry using a scintillation counter. Measurements were performed in triplicate and each experiment was performed using at least three independent skeletal muscle preparations.

### Statistical analysis

Data are expressed as the mean ± SD where *n* = 3 or the mean ± SEM where *n* ≥ 4. Differences between mean values were assessed using Student's *t* test. *P* < 0.05 was considered statistically significant.

### Materials

All chemicals were purchased from VWR (Lutterworth, UK), Sigma‐Aldrich (UK) or as otherwise stated. All solutions were prepared in deionized water (Millipore, Feltham, UK) and those used in bilayer experiments were filtered through a membrane with 0.45 μm pore diameter (Millipore).

## Results

**Figure 1:**
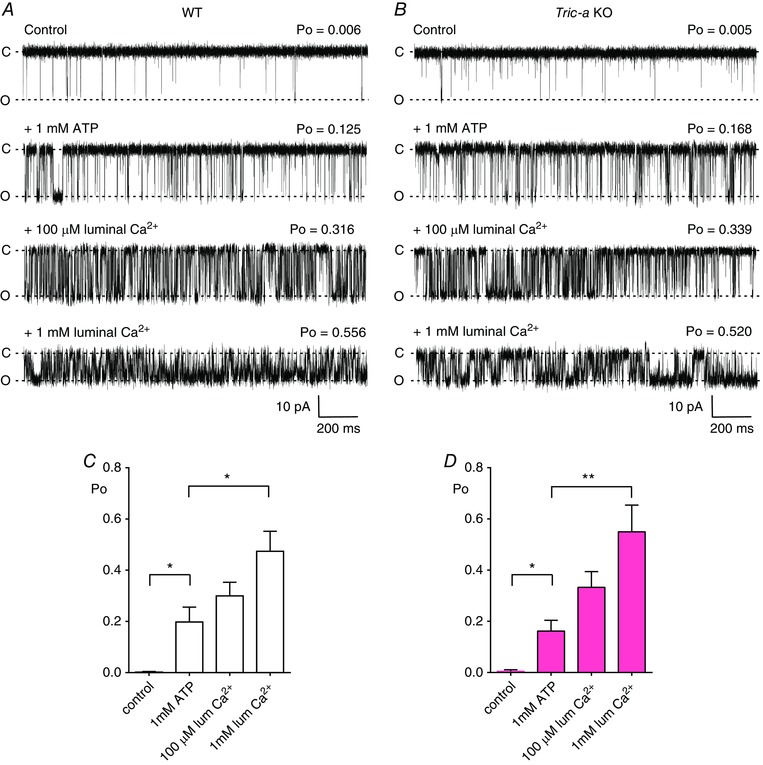
The effects of cytosolic ATP and luminal Ca^2+^ on RyR1 channels from *Tric‐a* KO mice with K^+^ as the permeant ion Representative single‐channel recordings of RyR1 from WT (*A*) and *Tric‐a* KO (*B*) mice under control conditions (10 μm cytosolic and luminal Ca^2+^), after subsequent addition of 1 mm cytosolic ATP (second traces) and subsequent increasing concentrations of luminal Ca^2+^ (100 μm and 1 mm as indicated). The bar charts below illustrate the mean data for RyR1 channels from WT (white) (*C*) and *Tric‐a* KO (pink) (*D*) skeletal muscle under control conditions, in the presence of ATP and increasing concentrations of luminal Ca^2+^. Values are the mean ± SEM (*n* = 6–10; ^*^ *P *< 0.05, ^**^ *P *< 0.01). The holding potential was −30 mV. O and C indicate the open and closed channel levels, respectively.

Evidence suggests that luminal Ca^2+^ is higher than normal in skeletal muscle from *Tric‐a* KO tissue (Zhao *et al*. 2010) yet Ca^2+^‐release is impaired, so we initially performed experiments using K^+^ as the permeant ion so that we could investigate whether the luminal Ca^2+^ sensitivity of the single RyR channels was modified. Figure 1, showing top traces from WT and *Tric‐a* KO tissue, demonstrates that the Po was similar for channels from both WT and *Tric‐a* KO tissue when the cytosolic and luminal [Ca^2+^] was maintained at 10 μm. Adding 1 mm ATP to the cytosolic channel side led to similar increases in Po in channels from WT and *Tric‐a* KO tissue (second trace), indicating that the response of RyR1 to ATP was not altered in *Tric‐a* KO tissue. Subsequently, increasing the luminal [Ca^2+^] to 100 μm and 1 mm also increased Po to levels that were comparable in both groups of channel, therefore providing no evidence for impairment of luminal Ca^2+^ sensitivity in RyR1 channels from *Tric‐a* KO tissue. Mean Po data are also shown (Fig. 1 *C* and *D*).

**Figure 2:**
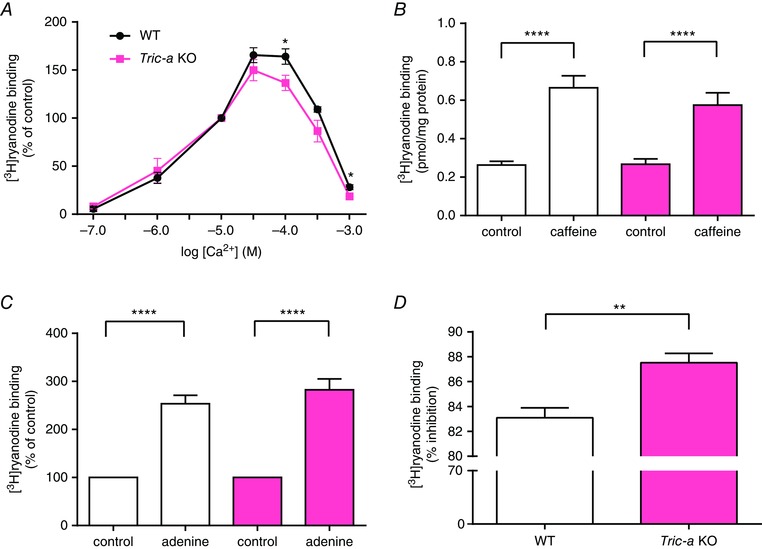
The effects of Ca^2+^, caffeine, adenine and Mg^2+^ on [^3^H]ryanodine binding to WT and *Tric‐a* KO skeletal muscle membrane vesicles (*A*), stimulation of [^3^H]ryanodine binding to WT and *Tric‐a* KO membrane vesicles by Ca^2+^. Free [Ca^2+^] was adjusted by EGTA and CaCl_2_ solutions according to the Maxchelator software (http://maxchelator.stanford.edu). Each point is the mean ± SEM (*n* = 7–9; ^*^ *P *< 0.05). Stimulation of [^3^H]ryanodine binding by caffeine (10 mm; *n* = 5 or 6) (*B*) and adenine (1 mm; *n* = 8) (*C*) was similar for WT and *Tric‐a* KO skeletal muscle membrane vesicles; *n* = 4; ^****^ *P *< 0.0001). The results in (*A*) and (*C*) are expressed as a percentage of the control binding at 10 μm Ca^2+^ for each genotype. (*D*), percentage inhibition of binding at 100 μm Ca^2+^ by 1 mm Mg^2+^. Mg^2+^ was significantly more effective at inhibiting [^3^H]ryanodine binding to skeletal muscle membrane vesicles from *Tric‐a* KO than from WT mice (*n* = 7; ^**^ *P *< 0.01). *Tric‐a* KO data are shown in pink.

We used [^3^H]ryanodine binding to the SR from WT and *Tric‐a* KO mice to examine the responses of populations of RyR1 channels in their native membranes to regulatory ligands (Fig. 2). The [Ca^2+^] concentration response relationship showed no difference in sensitivity to activating levels of Ca^2+^ but suggested that channels from *Tric‐a* KO mice are more sensitive to inhibition by high [Ca^2+^] (Fig. 2 *A*). Caffeine sensitizes RyR channels to activation by cytosolic Ca^2+^ (Holmberg & Williams 1990; Sitsapesan & Williams 1990) and we found that caffeine stimulated [^3^H]ryanodine binding to a similar extent in SR vesicles isolated from *Tric‐a* KO and WT tissue (Fig. 2 *B*). To examine the sensitivity of RyR to adenine nucleotides more thoroughly, we investigated the effects of adenine on [^3^H]ryanodine binding to SR vesicles. Adenine binds to the same sites as ATP on RyR channels (Rousseau *et al*. 1988; Chan *et al*. 2000; 2003) but cannot phosphorylate proteins and so the use of this compound allows an investigation of the response of the RyR channels to the direct effects of an agent binding to the adenine nucleotide‐binding sites on RyR without the complication of phosphorylation. SR vesicles contain a mix of many kinases that can be activated by ATP; thus, [^3^H]ryanodine binding studies cannot distinguish between the action of ATP as a reversible activator of RyR (by direct interaction with the adenine nucleotide binding sites on RyR) and the action of ATP with respect to inducing the phosphorylation of RyR or closely associated proteins. Adenine stimulated [^3^H]ryanodine binding to WT and *Tric‐a* KO SR to a similar extent (Fig. 2 *C*), confirming the results shown in Fig. 1 *A* and indicating that the interactions of ATP/adenine nucleotides with RyR are not altered in *Tric‐a* KO mice. We did find, however, that Mg^2+^ was significantly more effective at inhibiting the binding of [^3^H]ryanodine to *Tric‐a* KO SR than to WT SR (Fig. 2 *D*).

**Figure 3:**
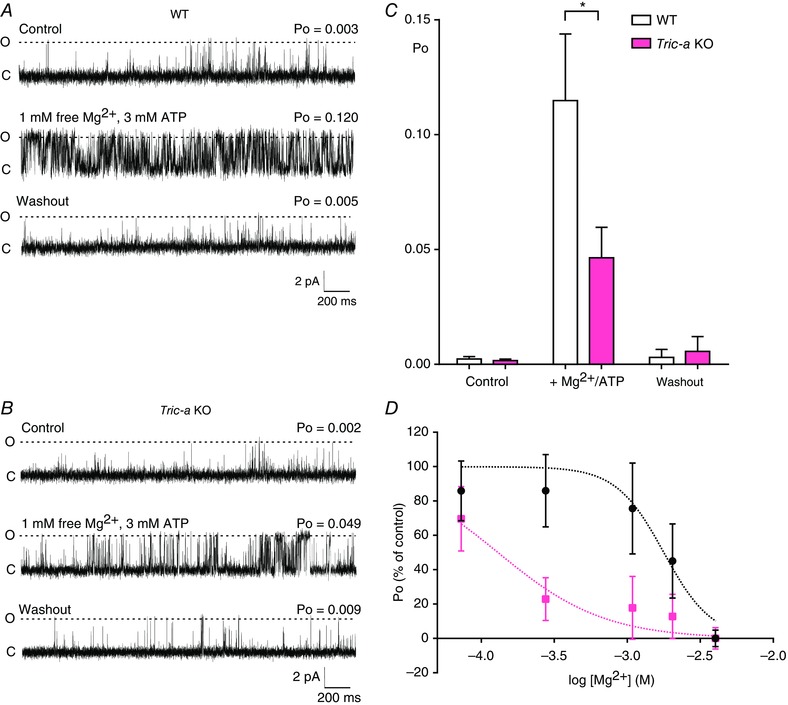
Comparison of the effects of Mg^2+^/ATP on the gating of RyR1 from WT and *Tric‐a* KO mice with Ca^2+^ as the permeant ion Representative RyR single‐channel recordings obtained from WT (*A*) and *Tric‐a* KO (*B*) mice under control conditions (top traces, 10 μm cytosolic Ca^2+^) in the presence of 1 mm free Mg^2+^/3 mm ATP/10 μm cytosolic Ca^2+^ (middle traces) and after washout of the Mg^2+^/ATP back to control conditions (bottom traces). The holding potential was 0 mV. O and C indicate the open and closed channel levels, respectively. *C*, mean Po values for RyR1 derived from WT (white) and *Tric‐a* KO mice (pink) under control conditions and in the presence of and after washout of Mg^2+^/ATP (*n* = 14 for WT; *n* = 22 for KO; ^*^ *P *< 0.05). *D*, single RyR1 channels from WT (black) or *Tric‐a* KO (pink) mice were activated with 10 μm cytosolic free Ca^2+^ and 1 mm ATP. The inhibition of Po with increasing concentrations of cytosolic Mg^2+^ was monitored and expressed as a percentage of the initial Po. Data points indicate the mean ± SEM Po for *n* = 12–18 for WT and *n* = 5–11 for *Tric‐a* KO channels. Dashed lines are the Hill equation fits to the data. IC_50_ values were 1.77 mm for WT and 0.13 mm for *Tric‐a* KO.

We therefore investigated whether altered regulation of RyR by Mg^2+^ was also manifest at the single‐channel level. With cytosolic Ca^2+^ as the sole activator, RyR Po is extremely low and variable, making comparisons of Mg^2+^ inhibition between the two groups of channels difficult and so we examined Mg^2+^ inhibition in the presence of ATP where Po is higher because we have shown that the response to adenine nucleotides is not affected in channels from *Tric‐a* KO mice. We used millimolar luminal Ca^2+^ (50 mm) as the permeant ion to induce optimum channel activity and the Po of RyR from WT and *Tric‐a* KO tissue with 10 μm free Ca^2+^ as sole activator was similar (Fig. 3 *A* and *B*, top traces), in agreement with the experiments with K^+^ as the permeant ion (Fig. 1 *A* and *B*). The representative traces show that the Po of RyR from *Tric‐a* KO mice was much lower than that of WT channels after adding 1 mm Mg^2+^ in the presence of 3 mm ATP, confirming the hypothesis that Mg^2+^ inhibition is more pronounced. Washout of Mg^2+^/ATP from the cytosolic chamber reversed Po back to control levels in both groups of channel (Fig. 3, bottom traces) demonstrating that the ATP‐induced increase in Po was not caused by phosphorylation of the channels by an endogenous kinase. The mean data are shown in Fig. 3 *C*.

To investigate whether the affinity of Mg^2+^ for RyR1 was altered in the *Tric‐a* KO mice, we activated single RyR1 channels with ATP first and then increased cytosolic [Mg^2+^]. The data shown in Fig. 3 *D* demonstrate that much lower concentrations of Mg^2+^ (WT: IC_50_ = 1.77 mm; *Tric‐a* KO: IC_50_ = 0.13 mm) were required to inhibit the channels from *Tric‐a* KO than from WT skeletal muscle, indicating that RyR1 affinity for Mg^2+^ is increased in *Tric‐a* KO skeletal muscle.

**Figure 4:**
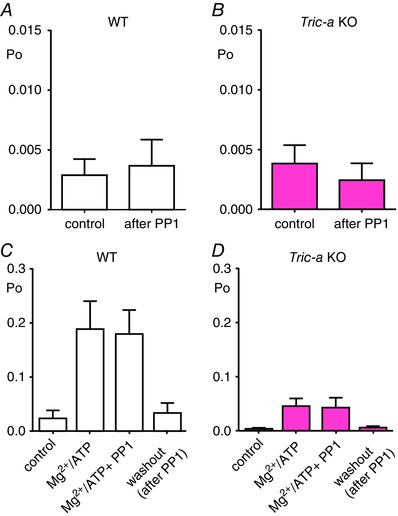
Effect of PP1 incubation on the gating of RyR1 from WT and *Tric‐a* KO mice with Ca^2+^ as the permeant ion RyR1 channel activity from WT (white) (*A*) and *Tric‐a* KO mice (pink) (*B*) in the presence of 10 μm cytosolic Ca^2+^ as sole channel activator before (control) and after 5 units of PP1 was added to the cytosolic chamber in the buffer from the supply company (New England Biolabs) for 10 min before washout of the cytosolic chamber back to control conditions in 10 μm cytosolic Ca^2+^ (after PP1). In addition, 5 units of PP1 was also added to RyR1 channels from WT (white) (*C*) and *Tric‐a* KO mice (pink) (*n* = 5) (*D*) in the presence of 1 mm free Mg^2+^/3 mm ATP/10 μm cytosolic Ca^2+^. The bar charts are labelled as control (10 μm cytosolic Ca^2+^ as sole channel activator), Mg^2+^/ATP (in the presence of 1 mm free Mg^2+^/3 mm ATP/10 μm cytosolic Ca^2+^), Mg^2+^/ATP + PP1 (5 units of PP1 was added to the cytosolic chamber in the buffer from New England Biolabs for 10 min in the presence of 1 mm free Mg^2+^/3 mm ATP/10 μm cytosolic Ca^2+^) and washout, after PP1 (10 μm cytosolic Ca^2+^ only) (*n* = 8 or 9).

**Figure 5:**
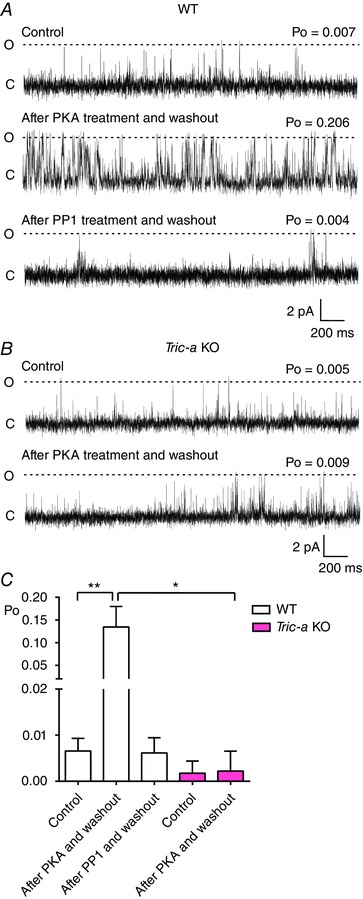
Effect of PKA‐dependent phosphorylation on the Ca^2+^‐dependence of gating of RyR1 from WT and *Tric‐a* KO mice with Ca^2+^ as the permeant ion RyR1 channel activity from WT (*A*) and *Tric‐a* KO (*B*) mice in the presence of 10 μm cytosolic Ca^2+^ as sole channel activator (Control, top traces), after washout of a 10 min treatment of 10 units of PKA, 3 mm ATP and 1 mm free Mg^2+^ (after PKA treatment and washout, second traces), and after washout of a 10 min treatment with 5 units of PP1 added to the cytosolic chamber in the buffer from New England Biolabs (after PP1 treatment and washout). Holding potential was 0 mV. O and C indicate the open and closed channel levels, respectively. *C*, mean Po of the RyR1 channels from WT (white) and *Tric‐a* KO mice (pink) under control conditions (control), after washout of PKA (after PKA and washout) and after washout of PP1 (after PP1 and washout) (*n* = 10–15; ^*^ *P *< 0.05, ^**^ *P *< 0.01).

It was previously reported that a change in Mg^2+^ inhibition of RyR1 can result when the channels are phosphorylated by PKA (Hain *et al*. 1994). We therefore examined whether there is a pre‐existing increased level of phosphorylation of RyR from *Tric‐a* KO tissue that could affect Po by investigating whether the phosphatase, PP1, could alter the gating of RyR from *Tric‐a* KO muscle. PP1 was added in buffer containing Mn^2+^, which could affect RyR activity. Therefore, after incorporation of channels into the bilayer with 10 μm Ca^2+^ as the sole channel activator, PP1 (5 units) was added to the cytosolic channel side and incubated for 10 min before washout of the PP1 and buffer back to control conditions (Fig. 4 *A* and *B*). There was no significant effect on channel gating either for channels from WT or *Tric‐a* KO tissue, indicating that no extra pre‐existing phosphorylation of these channels influenced the action of Mg^2+^/ATP on RyR from *Tric‐a* KO mice. The lack of effect of PP1 was not the result of an inadequate experimental protocol or inactive PP1 preparation because the same protocol was used to reverse the effects of phosphorylation of RyR (Fig. 5).

We also investigated whether PP1 could affect the reversible activation of RyR caused by Mg^2+^/ATP. Figure 4 *C* and *D* demonstrate that, in the presence of PP1, the blunted ability of RyR from *Tric‐a* KO muscle to respond to Mg^2+^/ATP is unchanged, providing further evidence that there is no pre‐existing altered phosphorylation state of RyR channels from *Tric‐a* KO mice that affects gating.

We next examined whether the response of RyR to phosphorylation was affected in mice devoid of *Tric‐a*. We recorded Po with 10 μm Ca^2+^ as the sole channel activator (Fig. 5). PKA (10 units), 3 mm ATP and 1 mm free Mg^2+^ were then added to the cytosolic chamber with the free [Ca^2+^] maintained at 10 μm. After 10 min of incubation, we then perfused away the PKA, Mg^2+^ and ATP back to the control conditions with 10 μm Ca^2+^ as sole activator. The typical response to phosphorylation is shown in the second traces. Note the irreversible increase in Po in the channels from WT mice, whereas the channels from *Tric‐a* KO mice exhibit no observable change in Po compared to controls. Figure 5 *C* shows the mean data. To test whether the increased Po occurring after PKA incubation was a result of phosphorylation of the channels, we added PP1 to the cytosolic chamber. PP1 reversed the actions of PKA incubation, demonstrating that the activation caused by PKA in WT channels was indeed caused by phosphorylation.

**Figure 6:**
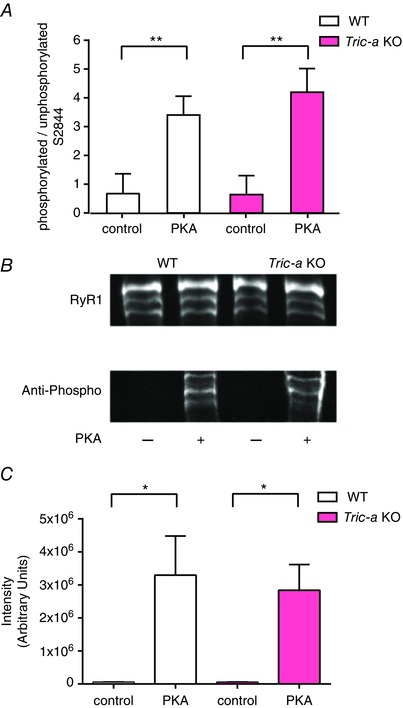
Comparison of phosphorylation of RyR1 from WT and *Tric‐a* KO mice before and after PKA treatment (*A*), ratio of phosphorylated: unphosphorylated MS intensities of RyR1 peptides containing S2844 for WT and *Tric‐a* KO before and after PKA treatment (*n* = 3 preparations for WT and *n* = 3 preparations for *Tric‐a* KO; ^**^ *P *< 0.01). (*B*), representative immunoblots of immunoprecipitated RyR1 showing total RyR1 and PKA‐dependent phosphorylation of RyR1 in WT and *Tric‐a* KO mice. (*C*), quantification of the anti‐phospho antibody signal relative to the total RyR1 signal (34C antibody) by densitometry of the immunoblots shown in (*B*). Data are from four independent experiments and are presented as the mean ± SEM (^*^ *P *< 0.05).

There is no commercially available antibody that recognizes specific phosphorylatable residues of RyR1. Accordingly, to investigate whether RyR1 from *Tric‐a* KO mice show abnormal levels of phosphorylation, we used MS to identify specific phosphorylated residues and a general phospho‐(Ser/Thr) PKA substrate antibody to observe any changes to phosphorylation of the RyR1 protein. MS identified RyR1‐Ser2844 as being phosphorylated and Fig. 6 *A* shows that the ratio of phosphorylated:non‐phosphorylated peptides containing Ser2844 was similar for WT and *Tric‐a* KO samples under basal conditions and was similarly increased following incubation with PKA. The general anti‐phospho‐(Ser/Thr) antibody also detected PKA‐dependent increases in phosphorylation that were similar for WT and *Tric‐a* KO samples (Fig. 6 *B* and *C*).

**Figure 7:**
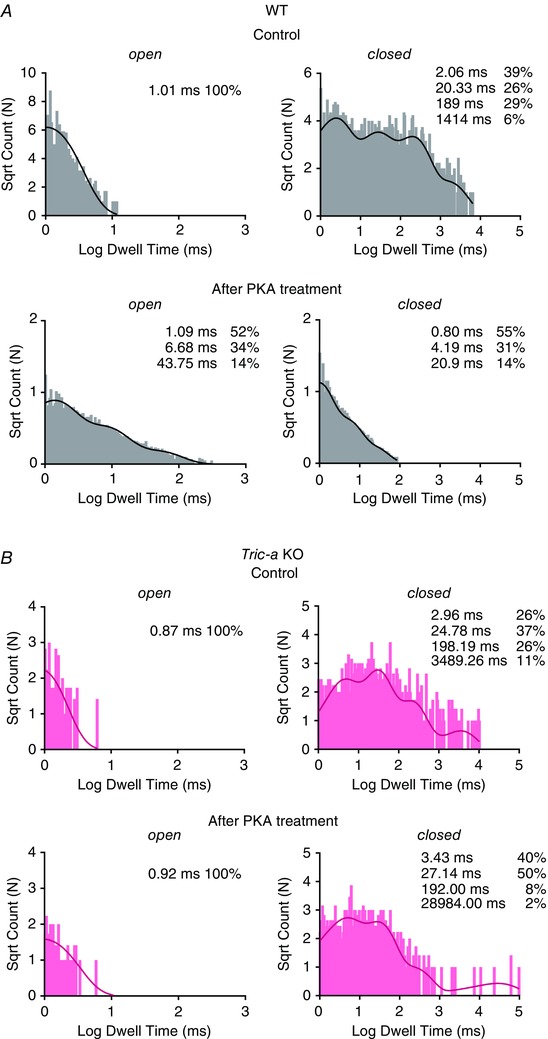
Effects of PKA‐dependent phosphorylation on open and closed lifetime distributions The open and closed lifetime distributions and pdfs for a typical single RyR1 channel from WT (grey) (*A*) and *Tric‐a* KO mice (pink) (*B*) in the presence of 10 μm cytosolic Ca^2+^ as sole channel activator before and after 10 min of treatment with 10 U of PKA. The best fits to the data were obtained by the method of maximum likelihood and the resulting time constants and percentage areas are shown.

In the experiments where only a single RyR was gating in the bilayer, lifetime analysis could be performed to investigate the mechanism by which phosphorylation increased Po. With cytosolic Ca^2+^ as sole channel activator, the main mechanism for increasing RyR1 Po is an increase in frequency of channel opening, with little change in the duration of the open states (Smith *et al*. 1986); hence, an agent that sensitizes the channel to cytosolic Ca^2+^ will primarily increase the frequency of channel opening. Before incubation with PKA, with 10 μm cytosolic Ca^2+^ as sole activator, there were few events and the open lifetimes were always extremely brief. Mean open times were similar for WT (0.47 ± 0.03 ms, SEM, *n* = 16) and for RyR from *Tric‐a* KO (0.46 ± 0.04 ms, SEM, *n* = 10) mice. Mean closed times were more variable (i.e. because the frequency of opening determines Po under these conditions) but were also comparable: 202.4 ± 84.9 ms (SEM, *n* = 14) for WT and 304.8 ± 124.4 ms (SEM, *n* = 5) for RyR from *Tric‐a* KO mice. It is interesting that, although there were no significant changes in the mean open (0.56 ± 0.13 ms, SEM, *n* = 6) or closed (101.2 ± 54.6 ms, SEM, *n* = 5) times for the RyR from *Tric‐a* KO mice after incubation with PKA, the mean open time of the channels from WT mice was increased significantly (1.47 ± 0.40 ms, SEM, *n* = 12, *P *< 0.05) with a non‐significant trend towards a reduction in mean closed time (46.12 ± 21.26 ms, SEM, *n* = 12). We investigated these changes further with lifetime analysis. The representative open and closed lifetime distributions of a typical RyR channel derived from WT mice, before and after phosphorylation by PKA are illustrated in Fig. 7. Table 1 shows the time constants and areas of the pdfs fitted to the distributions for all the single channels. We found that phosphorylation of RyR channels from WT mice led to a change in the distribution of open times, such that longer openings were observed and additional long open time components were resolved. The closed lifetime distributions shifted towards an increased proportion of short closings reflecting the increased frequency of opening. No changes in lifetime distributions were observed in the channels from *Tric‐a* KO mice following incubation with PKA (Fig. 7). As the significant change in gating behaviour observed with RyR from WT mice was an increase in the duration of open lifetimes, this suggests that phosphorylation does not just simply sensitize the channels to cytosolic Ca^2+^ (where we would observe little change in open lifetime durations) but that the channel can open in a Ca^2+^ independent manner, similar to the effects of PKA phosphorylation for RyR2 from cardiac muscle (Carter *et al*. 2011). This effectively means that the phosphorylated RyR channels derived from WT skeletal muscle possess an additional mechanism for channel activation that is independent of (and additional to) the cytosolic [Ca^2+^], whereas the RyR from *Tric‐a* KO mice do not.

**Table 1:** The effect of PKA‐dependent phosphorylation on lifetime parameters

	Open						Closed							
	T1 (ms)	A1 (%)	T2 (ms)	A2 (%)	T3 (ms)	A3 (%)	T1 (ms)	A1 (%)	T2 (ms)	A2 (%)	T3 (ms)	A3 (%)	T4 (ms)	A4 (%)
Control WT														
Ch. 1	0.67	100	–	–	–	–	0.53	33	2.90	32	23.87	26	132.00	9
Ch. 2	1.01	100	–	–	–	–	2.06	39	20.33	26	189.00	29	1414.00	6
Ch. 3	0.77	100	–	–	–	–	0.50	31	12.39	24	135	35	1370	9
Ch. 4	0.91	100	–	–	–	–	2.15	72	14.26	28	375.42	1	–	–
Control *Tric‐a* KO														
Ch. 1	0.78	100	–	–	–	–	1.45	27	17.31	27	172.56	29	1613.32	17
Ch. 2	0.87	100	–	–	–	–	2.96	26	24.78	37	198.19	26	3489.26	11
Ch. 3	1.42	100	–	–	–	–	2.29	34	19.22	39	152.31	27	–	–
Ch. 4	1.68	100	–	–	–	–	1.88	40	16.55	37	142.73	16	5789.07	7
PKA WT														
Ch. 1	1.09	52	6.68	34	43.7	14	0.80	55	4.19	31	20.9	14	–	–
Ch. 2	1.1	89	7.12	11	–	–	1.31	65	6.17	32	41.60	3	–	–
Ch. 3	0.65	80	3.71	18	35.9	2	0.73	53	3.51	32	19.88	14	731	1
Ch. 4	0.84	95	6.73	5	–	–	0.95	29	10.31	21	60.74	31	303.32	19
Ch. 5	0.8	94	5.95	6	–	–	1.26	80	7.32	19	342	1	–	–
PKA *Tric‐a* KO														
Ch. 1	0.81	100	–	–	–	–	4.36	54	41.26	30	369.00	9	–	–
Ch. 2	0.92	100	–	–	–	–	3.43	40	27.14	50	192	8	28984	2
Ch. 3	0.51	92	3.18	8	–	–	1.63	50	8.95	41	69.22	8	2533	1

Time constants (T1, T2, T3, T4) and percentage areas (A1, A2, A3, A4) are shown as obtained from maximum likelihood fitting of pdfs to open and closed lifetime distributions of single RyR channels from WT and *Tric‐a* KO muscle before and after PKA‐dependent phosphorylation.

**Figure 8:**
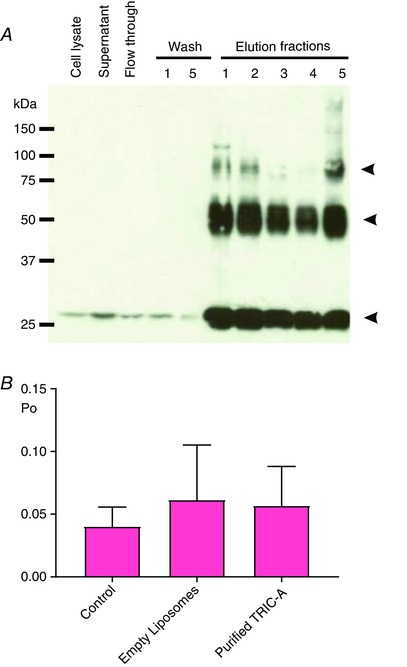
The effects of adding back purified TRIC‐A to the single‐channel function of RyR1 from *Tric‐a* KO mice (*A*), immunoblot illustrating the purification of recombinant TRIC‐A overexpressed in Chinese hamster ovary (CHO) cells. Lanes, from the left: crude cell lysate, supernatant containing solubilized TRIC‐A, PA‐tag column flow through, first and fifth washing steps of the PA‐tag column and all five elution fractions containing recombinant TRIC‐A protein. TRIC‐A protein from ‘Elution 5’ was reconstituted into phosphatidylcholine liposomes for the bilayer experiments. The membrane was probed with an anti‐PA tag antibody. The arrowheads indicate the monomeric, dimeric and trimeric form of TRIC‐A. (*B*), Po of RyR1 channels from *Tric‐a* KO mice in the presence of 1 mm free Mg^2+^/3 mm ATP/10 μm cytosolic free Ca^2+^ (Control, *n* = 17), after addition of empty phosphatidylcholine liposomes (empty liposomes, *n* = 8), or after addition of phosphatidylcholine liposomes containing purified TRIC‐A (purified TRIC‐A, *n* = 9).

We next performed a series of experiments where we added back purified TRIC‐A after incorporating RyR1 from *Tric‐a* KO skeletal muscle into bilayers to investigate whether the RyR1 response to Mg^2+^/ATP could be reversed back to that of the WT RyR1 channels. Figure 8 shows that this did not produce any significant increase in RyR1 Po, suggesting that Mg^2+^ inhibition was not relieved.

## Discussion

The results of the present study demonstrate that the RyR channels derived from *Tric‐a* KO skeletal muscle exhibit specific gating abnormalities. In the absence of Mg^2+^, the response of RyR from *Tric‐a* KO skeletal muscle to activating cytosolic ligands such as Ca^2+^, ATP, adenine and caffeine does not appear to be altered, nor is the sensitivity to luminal [Ca^2+^] affected. However, two specific abnormalities can be observed. First, Mg^2+^ exerts a greater inhibitory effect on RyR from *Tric‐a* KO skeletal muscle than on RyR from WT muscle as indicated by both [^3^H]ryanodine binding and single‐channel experiments. The [^3^H]ryanodine binding experiments also indicate that RyR from *Tric‐a* KO tissue is more readily inhibited by high [Ca^2+^] without any significant change in sensitivity to activation by low [Ca^2+^]. It therefore appears that the channels have become more sensitive to inhibition via the low affinity divalent cation binding sites.

The second major abnormality is that PKA, in the presence of Mg^2+^/ATP, can phosphorylate RyR derived from WT mice causing an increase in Po, although there is no increase in the Po of RyR from *Tric‐a* KO mice, even though the phosphorylation levels of both groups of channels are similar before and after PKA‐dependent phosphorylation (Fig. 6). In the RyR from WT mice, we were able to distinguish the activating effects of phosphorylation from the reversible effects of the ATP present in the incubation medium because, even with washout of the Mg^2+^/ATP/PKA back to the control conditions, where 10 μm Ca^2+^ is sole activator (Fig. 5), Po remained high. In all cases, incubation with PP1 then reversed the increase in Po back to control levels, confirming that phosphorylation was the cause of increase in Po. Thus, phosphorylation of the channels from *Tric‐a* KO mice cannot be translated into an increase in Po.

The altered functional properties of the RyR from *Tric‐a* KO mice that we describe could provide an explanation for the disrupted skeletal muscle function that is prevalent in *Tric‐a* KO mice (Zhao *et al*. 2010). A pathologically high level of SR Ca^2+^ is indicated by electron dense Ca^2+^ deposits within the SR and the high proportion of large vacuoles that are present (Zhao *et al*. 2010). The high SR Ca^2+^ content was confirmed by the use of caffeine, which caused a larger Ca^2+^ transient from flexor digitorum brevis fibres isolated from *Tric‐a* KO mice (Zhao *et al*. 2010). The reduced ability of RyRs to respond to activators or phosphorylation that we observe could lead to increased levels of SR Ca^2+^ because the release process would be markedly inhibited. This could also explain the reduced frequency of Ca^2+^ sparks observed in the muscle cells from *Tric‐a* KO mice (Zhao *et al*. 2010). Because Zhou *et al*. 2004 have shown that increasing [Mg^2+^] causes a reduction in spark frequency in mammalian skeletal muscle cells (Zhou *et al*. 2004), the increased inhibitory effect of Mg^2+^ on RyR opening that we observe in channels from *Tric‐a* KO mice would be expected to reduce spark frequency. In the presence of Mg^2+^, ATP is significantly more effective at reducing the mean closed time of RyR from WT (mean closed time = 7.72 ± 3.38 ms; *n* = 14) than from *Tric‐a* KO mice (mean closed time = 90.22 ± 30.46 ms; *n* = 10; *P *< 0.01). This indicates that the frequency of opening of RyR from *Tric‐a* KO mice does not increase effectively in response to ATP, a factor probably influencing spark number. Accordingly, as the level of Ca^2+^ within the SR becomes excessively high, and, presumably, because luminal Ca^2+^ sensitivity does not appear to be affected, this could lead to the reported ‘alternans’ contractile behaviour in tetanic stimuli to *Tric‐a* KO skeletal muscle (Zhao *et al*. 2010). If the luminal [Ca^2+^] content rises sufficiently high, the increased positive influence of luminal Ca^2+^ on RyR Po could just outweigh the extra Mg^2+^ inhibition and failure of the channels to respond to phosphorylation. The higher SR/cytosol Ca^2+^ concentration gradient would also lead to increased Ca^2+^ flux during each RyR opening, thus initiating the alternans pattern. In line with this thinking, it is interesting that, although we observed dampened activity of RyR1 and increased SR Ca^2+^ content in the skeletal muscle of *Tric‐a* KO mice, the opposite effect on SR Ca^2+^ content was observed in a mouse model with the malignant hyperthermia mutation Y522S (Manno *et al*. 2013). This mutation causes leaky RyR1 channels and the measured resting SR Ca^2+^ content in the malignant hyperthermia skeletal muscle cells was much lower than in the WT cells.

**Figure 9:**
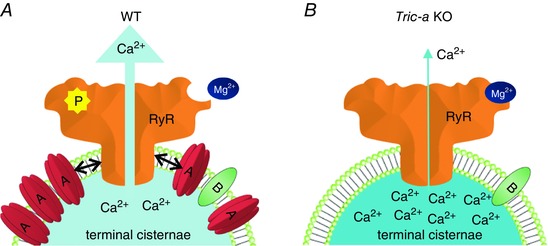
Model of proposed TRIC‐A modulation of RyR in skeletal muscle The terminal cisternae membrane of WT skeletal muscle (*A*) is densely packed with TRIC‐A and RyR (approximate ratio of 1:5:1 for RyR tetramer:TRIC‐A trimer:TRIC‐B trimer) providing ample opportunity for physical interactions between TRIC‐A and RyR. *Tric‐a* KO terminal cisternae membranes (*B*) are less sparsely populated with ion channels. The presence of TRIC‐A in the terminal cisternae of skeletal muscle causes conformational changes to RyR that promote dissociation of Mg^2+^, thus relieving the Mg^2+^‐induced suppression of the frequency of RyR channel opening. In the absence of TRIC‐A, Mg^2+^ inhibition of RyR is more pronounced and physiological activators such as ATP and luminal Ca^2+^ are less effective. Additionally, β‐adrenergic activation of PKA would not lead to an increase in RyR Po, as would occur in WT muscle.

There is widespread opinion that SR K^+^ channels provide the necessary charge compensation to fully balance the rapid loss of Ca^2+^ from the SR during the Ca^2+^ release process in skeletal muscle (Miller & Rosenberg 1979; Somlyo *et al*. 1981; Fink & Veigel 1996). This possible role was first suggested as early as 1979 (Miller & Rosenberg 1979). More recently, mathematical modelling of the ionic fluxes through RyR channels indicated that K^+^ currents through RyR should be amply able to compensate for the charge movements carried by Ca^2+^ during the Ca^2+^ release process (Gillespie & Fill 2008). Thus, the SR K^+^ channels may serve to balance monovalent cation concentration across the SR without majorly contributing to charge compensation. The results of the present study cannot shed light on the degree of counter‐current contributed by SR K^+^ channels, although they suggest that there may be additional mechanisms by which they could influence SR Ca^2+^ release. TRIC‐B is present in most cells at low levels but TRIC‐A is found at high levels in cardiac and skeletal muscle (Yazawa *et al*. 2007). It is calculated that, for every RyR1 in the junctional regions of skeletal muscle cells, there are approximately five TRIC‐A and one TRIC‐B channels (Pitt *et al*. 2010; Zhao *et al*. 2010). Thus, RyR and TRIC channels will jostle together in close proximity, allowing an opportunity for direct physical interactions that could modulate RyR gating in a reversible and dynamic manner. Figure 9 provides a model of the possible organisation of SR cation channels within the terminal cisternae and illustrates the void left by knocking out *Tric‐a*. In *Tric‐a* KO skeletal muscle, there is no evidence for up‐regulation of TRIC‐B to compensate (Venturi *et al*. 2013). At this point, it is worth considering that experiments performed in vascular smooth muscle cells and in HEK293 cells overexpressing RyR2 suggest that TRIC‐A modulates RyR channels, whereas TRIC‐B regulates inositol‐trisphosphate receptor channels (Yamazaki *et al*. 2011; Zhou *et al*. 2014). However, when we added back purified TRIC‐A channels after incorporating RyR1 from *Tric‐a* KO skeletal muscle into bilayers, the RyR1 response to Mg^2+^/ATP was not reversed back to that of the WT RyR1 channels (Fig. 8). It is possible that the interactions between TRIC‐A and RyR1 in the bilayer are delicate and require additional linking proteins or specific lipids. For example, the recently published structures of the *Caenorhabditis elegans* TRIC‐B channel (Yang *et al*. 2016), the bacterial TRIC protein (RsTRIC from *Rhodobacter sphaeroides*) and archaeal TRIC protein (SsTRIC from *Sulfolobus solfataricus*) (Kasuya *et al*. 2016) show that different lipids are integrated into the channels in different positions depending on the isoform.

In *Tric‐a* KO mice, RyR has only the possibility to interact with TRIC‐B (rather than TRIC‐A), although it is feasible that TRIC‐B cannot influence RyR function, as suggested previously (Zhou *et al*. 2014), or that any interactions influence RyR gating differently. Our experiments shown in Fig. 1 were conducted with K^+^ as the permeant ion. Recording RyR current fluctuations in this manner is not easy because of the multiple K^+^ channels that fuse with the bilayer and the excessive K^+^ currents can break the bilayer. Because it is rare to observe native RyR channel current fluctuations without simultaneous SR K^+^ channel currents, we can assume that, in those experiments where Ca^2+^ is the permeant ion (and so we cannot visualize K^+^ channel openings), there are probably also many K^+^ channels present in the bilayer that could influence RyR channel function. It is worth noting that there will be no ionic currents flowing through the many SR K^+^ channels (even when they open) in these experiments and so ionic flux through SR K^+^ channels cannot be a factor influencing RyR channel opening (unlike in the SR *in situ*) and, because the membrane is voltage clamped, counter‐current movement is irrelevant.

In summary, the experiments conducted in the present study suggest that TRIC proteins may influence RyR channel behaviour in ways additional to providing movement of monovalent cation current across the SR. The model shown in Fig. 9 summarises the potential influence of TRIC‐A on RyR gating and Ca^2+^ homeostasis in skeletal muscle. TRIC channels are expressed in high levels in the junctional SR and nuclear membranes and may affect the molecular architecture of these organelles and/or functionally interact with nearby proteins to directly influence SR Ca^2+^ movements. Thus, the TRIC proteins may influence SR Ca^2+^ movements by multiple mechanisms and further investigations are required to delineate the full extent of the interactions of TRIC‐A and TRIC‐B with closely positioned proteins.

## Additional information

### Competing interests

The authors declare that they have no competing interests.

### Author contributions

TI, MN and HT produced and characterized *Tric‐a* KO mice and provided tissue. SE, EV, MB, ADW, CL, DE and FO'B performed the single‐channel experiments. SE and EV analysed data and produced the figures. KW performed the [^3^H]ryanodine binding experiments and analysed the immunoblots. SE, EV, KW and DE isolated SR membrane vesicles. RS wrote the article. All authors discussed the results and commented on the article. All authors approved the final version of the manuscript submitted for publication.

### Funding

This work was supported by the British Heart Foundation (RG/10/14/28576, PG/13/76/30353, FS/11/31/28790 and awards through the BHF Oxford 4‐Year Studentship scheme), the Oxford BHF Centre of Research Excellence (RE/08/004), and the Japan Society for the Promotion of Science (Core‐to‐core program).
